# Predicting forest floor and woody fuel consumption from prescribed burns in southern and western pine ecosystems of the United States

**DOI:** 10.1016/j.dib.2017.10.029

**Published:** 2017-10-28

**Authors:** S.J. Prichard, M.C. Kennedy, C.S. Wright, J.B. Cronan, R.D. Ottmar

**Affiliations:** a School of Environmental and Forest Sciences, University of Washington, Seattle, WA, USA; b Sciences and Mathematics Department, University of Washington, Tacoma, WA, USA; c US Forest Service Pacific Northwest Research Station, Seattle, WA, USA

## Abstract

We present pre-burn biomass and consumption data from 60 prescribed burns in the southeastern and western United States. The datasets include pre-burn biomass in Mg/ha by fuel category: herbaceous fuels, shrubs, 1-hr, 10-hr, 100-hr, 1000-hr, 10,000-hr, and > 10,000-hr downed wood, litter and duff. Pre-burn depth (cm) and reduction (cm) are provided for litter and duff layers. Day-of-burn fuel moistures and weather are also listed by site.

**Specifications table**

**Table:** 

Subject area	*Forest Ecology*
More specific subject area	*Fire and fuels management*
Type of data	*Tables (csv format)*
How data was acquired	*Field and laboratory measurements*
Data format	*Summarized by site*
Experimental factors	*n/a*
Experimental features	In both regions, surface fires were burned within prescription windows detailed in site-specific burn plans. Fuel consumption was measured as the difference between sampled pre- and post-burn biomass
Data source location	*Southern sites: Florida (Eglin Air Force Base, Apalachicola National Forest, Saint Marks National Wildlife Refuge, and Pumpkin Hill Creek Preserve State Park) and South Carolina (Greenwood Preserve).*
	*Western sites: Arizona (San Carlos Apache Indian Reservation, Coconino and Tonto National Forests), Idaho (Wallowa-Whitman National Forest), Montana (Lubrecht Experimental Forest), Oregon (Crater Lake National Park, Deschutes, Malhuer and Ochoco National Forests) and Washington (North Cascades National Park, Okanogan-Wenatchee and Umatilla National Forests).*
Data accessibility	*Data are available within this article and will also be made available on the Forest Service Research Data Archive.*
Related research article	*Prichard, S.J., Kennedy, M.C., Wright, C.S., Cronan, J.B. and Ottmar, R.D., In press. Predicting forest floor and woody fuel consumption from prescribed burns in southern and western pine ecosystems of the United States. Forest Ecology and Management.*

**Value of the data**

• Reliable estimates of biomass and fuel consumption by fuel category (e.g., shrub, herb, downed wood by time lag class, litter and duff) are important to identify sources of smoke production. These data may be used in developing effective smoke reduction techniques and prescribed burn windows for wildland fire management.
• Site-specific pre-burn biomass and consumption data can be used in the development and testing of future fuel consumption models to be used for wildland fire planning.
• Data can also be used to validate biomass and carbon maps for the region.

## 1 Data

Predictive models are presented in a companion, full-length research article [1]. This article presents the source data from 60 prescribed burns in southeastern pine forests and 60 prescribed burns in western ponderosa pine pine-Douglas fir forests. Table 1 lists variables collected in this study and their definitions. Tables 2 and 3 present preburn and consumption values summarized by individual burn unit (hereafter referred to as sites). Table 4 lists comparison data compiled from published fuel consumption studies in the southern [2], [3], [4], [5], [6] and western [7], [8], [9], [10], [11], [12], [13], [14], [15] regions. Many of the western site comparison data were from broadcast burning of logging slash but fell within the distributions of unmanaged forests.

## 2 Experimental design, materials and methods

Fuel consumption during prescribed fires in southern pine forests were sampled during several field campaigns (Table 2) including 18 sites at Eglin Air Force Base in northwest Florida, 32 sites across northern Florida and in southern Georgia [16], and 10 additional sites in northern Florida [17]. Dominant overstory trees included longleaf pine (*Pinus palustris* Mill.), slash pine (*P. elliottii* Engelm.), sand pine (*P. clausa* (Chapm. ex Engelm.) Vasey ex Sarg.), loblolly pine (*P. taeda* L.), and pond pine (*P. serotina* Michx.).Understory vegetation included mesic flatwoods and sandhill forest or savanna and typically included saw palmetto (*Serenoa repens* (W. Bartram) Small), gallberry (*Ilex glabra* (L.) A. Gray), turkey oak (*Quercus laevis* Walter) and wiregrass (*Aristida stricta* Michx). Fires were generally ignited as strip head fires by using drip torches.

A total of 60 prescribed fires were sampled in ponderosa pine-dominated forests in Arizona, eastern Oregon, eastern Washington and Montana. Sites were selected to span a range of elevations but were confined to slope gradients less than 60 percent and where fuels were relatively homogenous. Dominant trees included ponderosa pine (*Pinus ponderosa* Douglas ex C. Lawson) and Douglas-fir (*Pseudotsuga menziesii* (Mirb.) Franco) with grass and mixed shrub understories. Ignition technique and pattern varied at the discretion of fire personnel and included ground ignition with drip torches and aerial ignition with exothermic spheres. Sites were generally unmanaged, but nine sites had been thinned prior to burning and contained scattered logging slash (Table 3).

In both regions, low- to moderate-intensity surface fires were burned within prescription windows detailed in site-specific burn plans. Fuel consumption was measured as the difference between sampled pre- and post-burn biomass in the following categories: shrubs, herbs (i.e., graminoids and forbs), downed wood by time lag class [18], litter and duff. Forest litter is defined as undecomposed dead plant matter that has fallen to the ground (i.e., the Oi soil horizon). Duff is defined as partially to fully decomposed litter (i.e., the Oa and Oe soil horizons). Downed wood time lag size classes are defined by diameter thresholds and include 1-hr (< 0.64 cm), 10-hr (0.64–2.54 cm), 100-hr (2.54–7.62 cm), sound large down wood (SLDW, > 7.62 cm) and rotten large downed wood (RLDW, > 7.62 cm).

Pre- and post-fire biomass were measured in sample plots and transects that were placed systematically along grids within areas with relatively uniform fuels and vegetation. A minimum of nine pre-burn and nine post-burn sampling grid points were established before each prescribed fire. Abrupt changes in vegetation or site discontinuities (e.g., steep slopes, rocky outcrops, and riparian areas) were avoided during plot setup.

At southern sites, fine surface fuels (i.e., shrubs, herbs, and fine downed wood (FDW, < 7.6 cm in diameter)) were inventoried using destructive sample plots. A minimum of nine pre-burn and nine post-burn clip plots were sampled within each inventory unit. Live and dead vegetation was clipped from within a square plot, bagged and returned to the laboratory, oven-dried at 100 °C for a minimum of 48 h until a constant weight was achieved and then weighed with a precision balance to determine dry-weight biomass [16], [19]. Shrubs were generally collected within 4-m^2^ square plots and included all live and dead shrub biomass that was rooted inside of the plot. Grasses, forbs, litter and duff were sampled within smaller plots (0.5–1-m^2^) nested within each shrub biomass plot. SLDW and RLDW were surveyed along 20 to 30-m long planar intersect transects [18] before and after each prescribed fire. Where large downed wood was abundant, we used an alternate technique for determining large woody fuel consumption. We measured diameter reduction on a randomly selected sample of 20 logs > 7.62 cm in diameter at least three meters in length that were randomly selected from the area covered by the gridded plot design. Prior to burning, a steel wire was wrapped and secured tightly at a perpendicular angle to the mid-point of a log such that the length of the wire became a measurement of pre-burn circumference. Following burning, each wire was pulled tightly around the remaining section of the log; the new length represented the post-burn circumference. Circumference was converted to diameter by dividing by pi, and the difference between measurements was used to calculate absolute and fractional diameter reduction. Fractional diameter reduction was multiplied by pre-burn biomass, which was derived from a planar intersect inventory, to calculate large downed wood consumption.

At western sites, shrubs and grasses were absent or uncommon on most sites and were not sampled. Large downed wood consumption was sampled using the same methods as for southern pine sites. However, fine wood was measured on planar intersect transects [18] before and after each fire instead of the fixed area plot sampling approach used in southern sites. The length of transect that was sampled was dependent on the fine wood size class, with transect length increasing with size class. Litter and duff consumption were measured as depth reduction using steel nails (hereafter referred to as pins) inserted vertically into the forest floor prior to the burn. Sixteen pins were placed systematically within two meters of the origin of the downed wood sampling transects. Each pin was inserted into the forest floor until embedded in mineral soil with the top of the pin flush with the forest floor surface. Litter depth around each pin was measured prior to the fire, taking care not to disturb or alter the litter. Following each fire, the length of the exposed pin and depth to mineral soil were measured. Litter depth reduction was calculated by subtracting the length of the exposed pin (up to the depth of the pre-burn litter) from the pre-burn litter depth. Pre-burn duff depth was calculated by subtracting pre-burn litter depth from the depth to the mineral soil, and duff reduction was calculated by subtracting the pre-burn litter depth from the length of the exposed pin. Pre-burn depths and post-burn reduction were multiplied by material-specific bulk density values to calculate litter and duff biomass and mass consumed.

Day-of-burn fuel moisture samples were collected prior to ignition at all sites. Samples of 10-hr and 100-hr downed wood, litter and duff were collected across the entire area within the burn unit covered by gridded plots and stored in heavy-gauge, air-tight plastic bags. Live fuels (e.g., grasses, forbs and shrubs) were also collected by lifeform to assess moisture content. Samples were weighed within eight hours of being collected, oven-dried at 100 °C for a minimum of 48 h until a constant weight was achieved and then reweighed with a precision balance determine gravimetric moisture content. For 1000-hr downed wood, each wired log (≥ 7.62 cm) was sampled for fuel moisture by sawing disks from near the small and large ends; samples were dried and weighed, as above. Individual log final fuel moisture was recorded as the average of the two disks.

### 2.1 Statistical analysis and model selection

Pre-burn fuel loading (Mg ha^−^^1^), fuel consumption (Mg ha^−^^1^), and gravimetric fuel moisture content (%) were summarized by fuel category (shrub, herb, downed wood by time lag class, litter and duff) for each inventory unit. Simple and multiple ordinary least squares (OLS) regression models were constructed in R [20]. Final models were selected based on lowest Aikake's Information Criterion (AIC) values and generally included pre-burn loading and fuel moisture as predictor variables. Because most models exhibited a mean-variance relationship in the residuals, we constructed final models and prediction intervals using weighted least squares regression with weights defined as 1/pre-burn load. Prediction intervals were calculated across the sampled range of pre-burn loading. For models that had a second predictor variable such as 1000-hr, litter or duff fuel moisture, the average of sampled values was used for prediction interval estimation.

## Footnotes

Appendix A Supplementary data associated with this article can be found in the online version at 10.1016/j.dib.2017.10.029.

## References

[1] Prichard S.J., Kennedy M.C., Wright C.S., Cronan J.B., Ottmar R.D. (2017). Predicting forest floor and woody fuel consumption from prescribed burns in southern and western pine ecosystems of the United States. For. Ecol. Manag..

[2] B.D. Clinton, J.M. Vosé, W.T. Swank, E.C. Berg, Fuel consumption and fire characteristics during understory burning in a mixed white pine-hardwood stand in the southern Appalachians. USDA For. Serv. Res. Pap. SRS-RP-12, 1998.

[3] E.R. Scholl, T.A. Waldrop, Photos for estimating fuel loadings before and after prescribed burning in the upper Coastal Plain of the Southeast. USDA For. Serv. Gen. Tech. Rep. GTR-SRS-26, 1999.

[4] Sullivan B.T., Fettig C.J., Otrosina W.J., Dalusky M.J., Berisford C.W. (2003). Association between severity of prescribed burns and subsequent activity of conifer-infesting beetles in stands of longleaf pine. For. Ecol. Manag..

[5] Kolaks J. (2004). Fuel Loading And Fire Behavior In The Missouri Ozarks Of The Central Hardwood Region (MS Thesis).

[6] Reid A.M., Robertson K.M., Hmielowski T.L. (2012). Predicting litter and live herb fuel consumption during prescribed fires in native and old-field upland pine communities of the southeastern United States. Can. J. For. Res..

[7] S.S. Sackett, Reducing Natural Ponderosa Pine Fuels Using Prescribed Fire: Two Case Studies, USDA For. Serv Res. Note. RM-RN-392, 1980.

[8] S.N. Little, F.R. Ward, D.V. Sandberg, Duff Reduction Caused By Prescribed Fire On Areas Logged To Different Management Intensities, USDA For. Serv. Res. Note. PNW-RN-397, 1982.

[9] J.K. Brown, M.A. Marsden, K.C. Ryan, E.D. Reinhardt, Predicting Duff And Woody Fuel Consumed By Prescribed Fire In The Northern Rocky Mountains, USDA For. Serv. Res. Pap. INT-RP-337, 1985.

[10] R.D. Ottmar, S.N. S.N. Little, J.L. Ohmann, Predicting duff reduction to reduce smoke from clearcut slash burns in western Washington and western Oregon, in: Proceedings of the Eighth Conference on Fire and Forest Meteorology, Detroit, Michigan, American Meteorological Society, 1985.

[11] R.D. Ottmar, Prescribed fire and fuel consumption in uncured slash – preliminary results, in: Proceedings of the Ninth Conference on Fire and Forest Meteorology, San Diego, California, Society of American Foresters, 1987.

[12] E.D. Reinhardt, R.E. Keane, J.K. Brown, First Order Fire Effects Model: FOFEM, USDA For. Serv. Gen. Tech. Rep. GTR-INT-344, 1997.

[13] Hille M.G., Stephens S.L. (2005). Mixed conifer forest duff consumption during prescribed fires: tree crown impacts. For. Sci..

[14] Knapp E.E., Keeley J.E., Ballenger E.A., Brennan T.J. (2005). Fuel reduction and coarse woody debris dynamics with early season and late season prescribed fire in a Sierra Nevada mixed conifer forest. For. Ecol. Manag..

[15] Agee J.K., Lolley M.R. (2006). Thinning and prescribed fire effects on fuels and potential fire behavior in an eastern Cascades forest, Washington, USA. Fire Ecol..

[16] Wright C.S. (2013). Fuel consumption models for pine flatwoods fuel types in the southeastern United States. South. J. Appl..

[17] Cronan J.B., Wright C.S., Petrova M. (2015). Effects of dormant and growing season burning on surface fuels and potential fire behavior in northern Florida longleaf pine (Pinus palustris) flatwoods. For. Ecol. Manag..

[18] J.K. Brown, Handbook For Inventorying Downed Woody Material, USDA For. Serv. Gen. Tech. Rep. INT-GTR-16, 1974.

[19] S.J. Prichard, C.S. Wright, R.E. Vihnanek, R.D. Ottmar, Predicting forest floor and woody fuel consumption from prescribed burns in ponderosa pine forests, in: Proceedings of the Third International Fire Ecology and Management Conference: Fire as a Global Process, San Diego, CA, 2006.

[20] Core Team R. (2016). R: A Language And Environment For Statistical Computing.

